# Dynamics of changes in motor development depending on the quality in the 3rd month of life

**DOI:** 10.3389/fpubh.2022.939195

**Published:** 2022-09-16

**Authors:** Ewa Gajewska, Mariusz Naczk, Alicja Naczk, Magdalena Sobieska

**Affiliations:** ^1^Department of Developmental Neurology, Poznan University of Medical Sciences, Poznan, Poland; ^2^Institute of Health Sciences, Collegium Medicum, University of Zielona Gora, Zielona Gora, Poland; ^3^Faculty of Physical Culture in Gorzow Wielkopolski, Poznan University of Physical Education, Pozna, Poland; ^4^Department of Rehabilitation and Physiotherapy, Poznan University of Medical Sciences, Poznan, Poland

**Keywords:** infant, motor development, qualitative assessment, 3rd month, quantitative assessment

## Abstract

The aim of the study was to show that the quantitative and qualitative motor development from the 3rd month of life is key to achieving milestones and that it may be an early warning signal in children at risk of cerebral palsy (CP). The study population included 93 children (69 born at term). Children were born at week 38 ± 4, the mean body weight was 3,102 ± 814 g. All children were evaluated after reaching the 3rd month of life (quantitative and qualitative assessment), and then the 4.5th, 7th, and 12th of life (quantitative assessment). In case of suspected CP, children were followed until the 18th month, when the diagnosis was confirmed. If at the age of 3 months, a child achieved a quadrangle of support and symmetrical support, then its development at the 4.5th month of life was correct, it would creep, and it would assume a crawl position, then in the final assessment (12th month of life), the child would start to walk. If a child failed to achieve a quadrangle of support and symmetrical support and the dynamics of its development were incorrect, the development would be delayed (12th month of life), or CP would develop. A correct qualitative assessment in the 3rd month of life with a high probability guarantees corrects quantitative development at the 4.5th, 7.5th, and 12th months of life. If the qualitative assessment in the 3rd month of life was very low the child would probably be diagnosed with CP at 18 months.

## Introduction

Motor behavior is especially based on spontaneous, patterned activity, which is a quintessential feature of neural tissue (1). Signs of motor delay or abnormal achievement of milestones may be caused by motor disorders or other developmental problems (2, 3). These symptoms may be non-specific, but early detection is essential for implementing an early intervention (4, 5). Timely interventions (physiotherapy) can prevent or minimize developmental delays and prevent unnecessary secondary effects of motor disorders (6–8). To facilitate early detection of motor problems and developmental delays, it is recommended to use standardized assessment methods that provide precious information compared to the clinical observation itself (9, 10). It seems that an assessment based solely on clinical observation can detect a smaller number of children with developmental disorders, and they are detected at a late stage, compared to the evaluation based on the use of standardized, validated scales or sheets (11, 12). The tools used to assess development should be validated and easy to apply, available, acceptable to the investigator and the subject, and should provide practical benefits. In routine medical practice in Poland, motor development is monitored by the child's achievement of milestones as a predetermined and sequential progression from one skill to another and by checking muscle tone or sometimes only reflexes. On the other hand, contemporary theories suggest that motor behavior should not be analyzed mainly in terms of reflexes (13). Motor development is a flexible and adaptive process and depends on continuous feedback between the brain, body, and environment. The observation of achieving milestones does not reflect this complex process of motor development. Moreover, milestones appear with great variability in the first year of life (14) and tell us nothing about what may underlie the delay in their achievement (15).

The assessment if they appeared on time (YES/NO or 1/0) does not allow to detect children with motor disorders early. On the other hand, the assessment of the quality of mobility, describing the way of performing movements, may help in the early detection of children with motor disorders, and thus avoid the loss of valuable time for intervention, which may be caused by waiting for the achievement of milestones (5, 16). Despite the global nature of locomotion patterns, their individual motor elements (quality), also known as partial movement patterns, are probably separately and individually registered by the central nervous system (CNS). This is due to the varied “timing” of memory processes in the CNS (17).

Many authors state that the 3rd month of life is significant for motor development, which is the moment of severe changes (1, 18). In the 3rd month of life, following elements of motor behavior should be present: a symmetrical position on the back in the supine position, bringing arms together in the centerline, lifting them above the ground in the area of the lower limbs, bent at the hip and knee joints with the pelvis in an intermediate position, with symmetrical support on elbows with isolated head rotation in the prone position (17, 19).

Historically, the successive movement components in the back-to-abdominal turning process were first discovered as kinesiological components of ideal motor development. The lack of these components in pathological development was logically treated as a blockage in motor ontogenesis. One of the components of the movement in turning from the back to the abdomen is turning to the side, which, according to Vojta, occurs when a baby reaches the 4.5th month of life. When a child performs an intended grip, his/her body's center of gravity is displaced laterally, the pelvis is slanting in the frontal plane, and the activity of the legs is differentiated. Asymmetrical stretching of the chest occurs, support on the underlying shoulder takes place, which is possible only if the direction of vectors of surrounding muscles is distal, and the underlying lower half of the chest is expanded (17). Turning to the side was also shown in the Motor Assessment of the Developing Infant (20), which similarly occurs in child development, between the 4th and the 5.5th month of life, while in a Hammersmith Infant Neurological Examination (HINE) it is described as one of the stages of motor development occurring at the 4th month of life (21). A component of development observed at the same age, i.e., at the 4.5th month of life in the prone position, is the asymmetric support on one elbow (17), i.e., when the point of support is moved to the side to one elbow during an attempt to make a grasp at a specific target. The head and grasping hand are raised beyond the support base. The grasping arm is bent at the shoulder joint at an angle of approximately 120 degrees. The child is supported on one elbow, on the hip joint located on the same side as the supporting elbow, and on the opposite knee, which is bent at an angle of approximately 90 degrees (17). A similar milestone of development was described more generally by Piper and Darrah (20), and the month of its occurrence was adopted as the period between the 5th and 7th month, while in the assessment according to the Hammersmith scale, this stage of development was not included (21). In our study, the age of turning to the side and the asymmetric support on one elbow was adopted as the 4.5th month of life following Vojta. The first form of forward movement is creeping, sometimes called “seal movement” by Vaclav Vojta (17), when a child moves on the abdomen mainly using the upper limbs, without lifting the pelvis above the ground. Simultaneously with this activity, a child begins to adopt the crawl position (raising the trunk over the surface, on extended upper limbs with open palms and knees), and it is preceded by high support on extended upper limbs with open palms and raising the chest high (22). These two functions are characteristic of a child aged about 7.5 months (17), while independent walking (moving quickly with short steps) is analyzed at 12 months. The age criterion was adopted following Piper and Darrah and Vojta (17, 20).

### Aim of the study

The objective of the study was to show that the quantitative and qualitative motor development from the 3rd month of life is key to achieving the milestones and that it may be an early warning signal in children at risk of cerebral palsy (CP).

### Additional aim

Symmetrical support in the 3rd month with high probability heralds the emergence of subsequent milestones on time.

## Materials and methods

The study sample consisted of children who raised no suspicion as to their motor development, born at term or preterm (between week 28 and 42) or children whose parents/caregivers asked for appointment at the Clinic of Neurology, Poznan, Poland for periodic assessment of the development with a referral from a general practitioner, a pediatrician or because of parents' concerns (weak head control in the traction response or suspicion of delayed development).

The entire study population included 93 children; 69 born at term and 24 born preterm, 50 boys and 43 girls. Children were born at week 38 ± 4 (born at term 39 ± 1/preterm 33 ± 3), the mean body weight was 3,102 ± 814 g (born at term 3,470 ± 428/preterm 2,044 ± 730). Preterm children were assessed at the corrected age.

Exclusion criteria were genetic or metabolic disorders, severe birth defects, or extreme preterm birth (below 28 gestation week). No children with microcephaly or macrocephaly were included.

All examined children were evaluated after reaching the 3rd month of life (12–16 weeks after birth) and then the 4.5th, 7th, and 12th month of life. In case of suspected CP, children were followed until the 18th month, when the diagnosis was confirmed.

The majority (64 children) were born vaginally, 23 by Cesarean section, four by forceps delivery, and two with a vacuum.

Considering the scoring according to the Apgar scale in the 5th minute of life, 88 infants were in good condition, four were born in a semi-severe condition, and one was born in severe condition. Bleeding into the brain's ventricles (IVH) occurred in nine children (IVH I°–two children, II°–three children, III°–three children, and IV°–one child). Respiratory distress syndrome occurred in nine children, hypotrophy and hyperbilirubinemia in two. In most children, these disorders occurred in preterm children; only one full-term child developed grade IV IVH.

### Procedure

#### Quantitative assessment in the 3^rd^ month of life

In all children, the physiotherapeutic qualitative assessment of motor performance at 3 months was performed in the prone and supine positions.

This assessment consisted of a quadrangle of support in the supine position (head in the axis of the body, upper limbs aim at the centerline, lower limbs flexed up to 90 degrees in the hip and knee joint, foot in an intermediate position), and symmetrical support on elbows in the prone position.

The functional assessment was performed by a physiotherapist, who assessed 3-month-old children (at least 12 weeks completed, in case of preterm babies, corrected age was considered), according to the previously described “Quantitative and qualitative assessment sheet” (16, 18, 22).

#### The qualitative assessment in the 3^rd^ month

A qualitative assessment was also made in the 3rd month of life, which included 15 in the supine and 15 elements in the prone position. In the supine position, the assessment involved: head symmetry, spine in extension, shoulder in a balance between external and internal rotation, wrist in an intermediate position, thumb outside, palm in an intermediate position, pelvis extended, lower limb situated in moderate external rotation and lower limb bent at the right angle at hip and knee joints, foot in intermediate position—lifting above the substrate. In the prone position, the assessment involved: isolated head rotation, arm in front, forearm in an intermediate position, elbow outside of the line of the shoulder, palm loosely open, thumb outside, spine segmentally in extension, scapula situated in medial position, pelvis in an intermediate position, lower limbs situated loosely on the substrate, foot in an intermediate position. Both sides were assessed for symmetrical parts of the body to exclude asymmetry.

Each element was assessed as 0—element performed only partially or entirely incorrectly, 1—element performed entirely correctly. The duration of the examination performed by the physiotherapist was between 10 and 15 min. Each assessed element had to be observed at least three to four times during the test. A maximum of 15 points could be given for the prone position and a maximum of 15 points for the supine position.

Interobserver reliability ranged from 0.870 to 1.000, while intraobserver reliability was equal to 1 (16). Previously, this type of examination was used in the assessment of children aged 3 months, and the comparison between physiotherapeutic and neurological assessment showed high agreement, with high conformity coefficients (*z* = −5.72483, *p* < 0.001) (16).

The examination was performed at the Center for Child and Adolescent Neurology Clinic in 2011–2016 following the ethical standards, the 1964 Helsinki declaration, and its later amendments. Children recruited for the study were patients/clients of the Child Neurology Center. All parents/caregivers agreed to participate in the study, as apart from routine assessment and therapy no extra visit was necessary.

#### The quantitative assessment at the age of 4.5 months

Subsequently, all children were assessed at 4.5 months; turning to the side was checked in the supine position, and asymmetrical support on one elbow was checked in the prone position (YES/NO).

#### The quantitative assessment at the age of 7.5 months

At 7.5 months, it was assessed whether a baby creeps and whether it assumes the crawl position (YES/NO).

#### The quantitative assessment at the age of 12 months

At 12 months, it was assessed whether a baby walks independently (moves quickly with short steps) (YES/NO).

Finally, children who achieved independent walking (walking at the age of 12 months), delayed and diagnosed as CP (suspected at 12 months, finally diagnosed at 18 months) are shown.

Then, children with suspected CP underwent a neurological assessment to verify the diagnosis at 18 months.

## Statistical methods

For statistical analysis, Statistica Software was used. The estimated elements were assessed as present/absent (1/0) and their sum represented an ordinary variable, thus, the results were presented as medians with quartiles (Me, Q25–Q75). The non-parametric tests were used. To compare two groups, the U Mann-Whitney test was used. To compare more groups Kruskal-Wallis ANOVA with the *post-hoc* Dunn's test was used. The assumed statistical significance level was *p* < 0.05.

## Results

**Figure 1:**
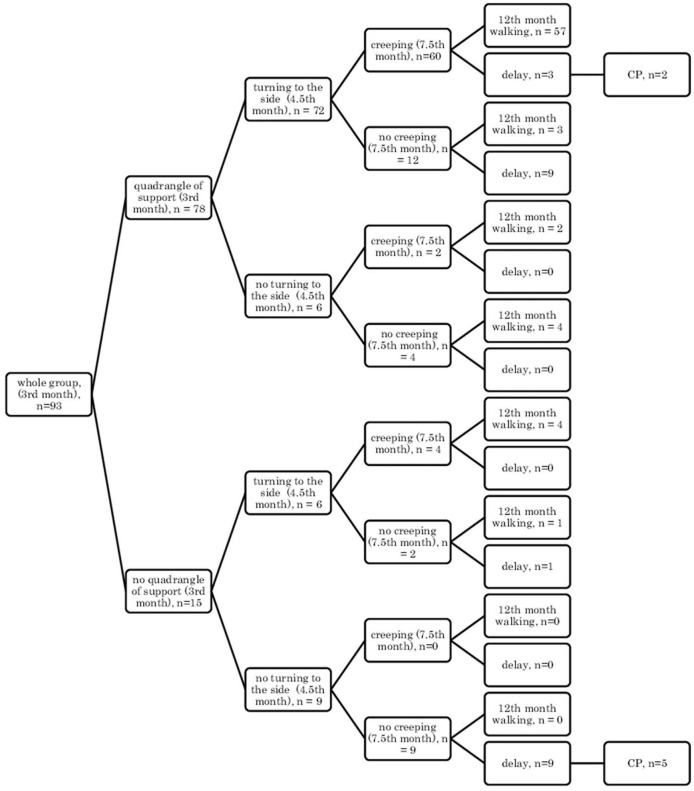
Motor development in the supine position: a quadrangle of support (3rd month); turning to the side (4.5th month); creeping (7.5th month), and independent walking (12th month).

**Figure 2:**
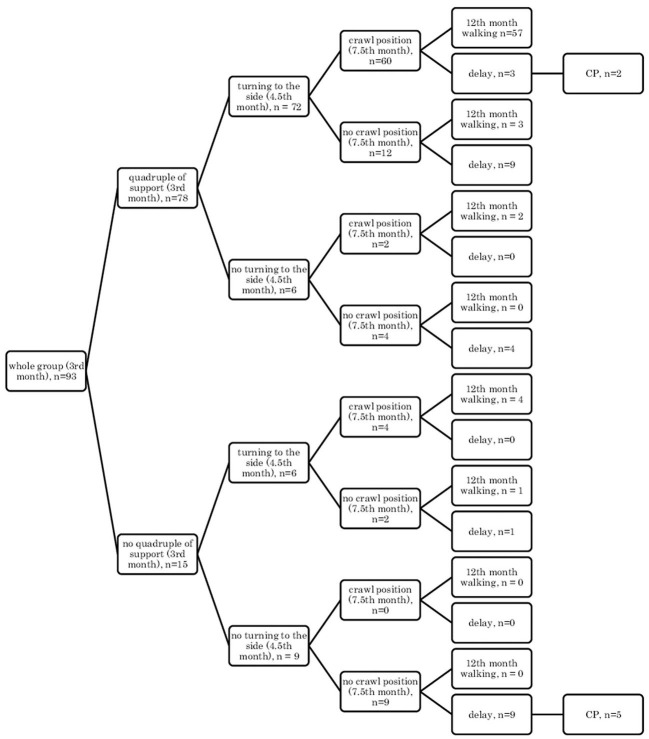
Motor development in the prone position: symmetrical support (3rd month); asymmetric support (4.5th month); creeping (7.5th month), and independent walking (12th month).

**Figure 3:**
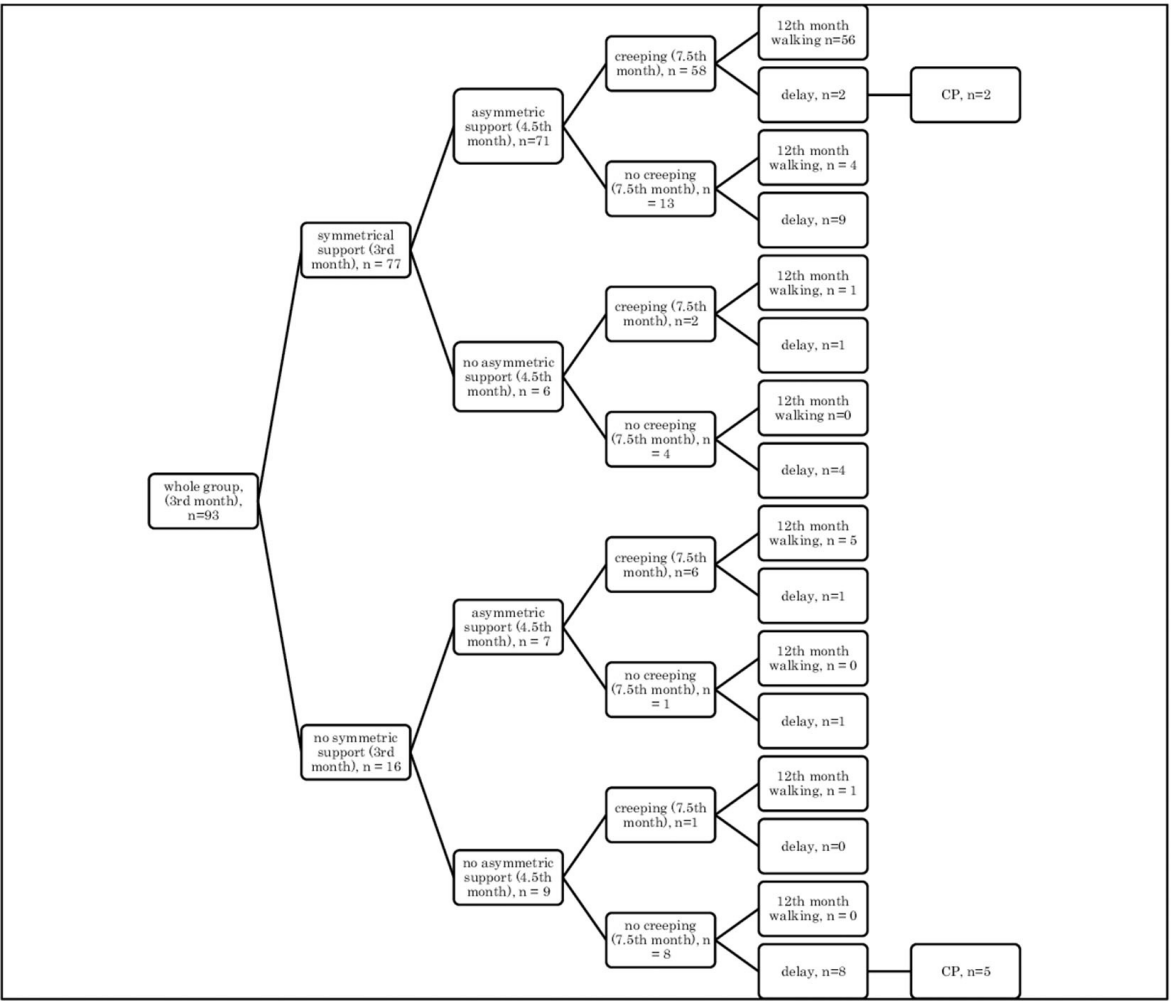
Motor development in the supine position: a quadrangle of support (3rd month); turning to the side (4.5th month); crawl position (7.5th month), and independent walking (12th month).

**Figure 4:**
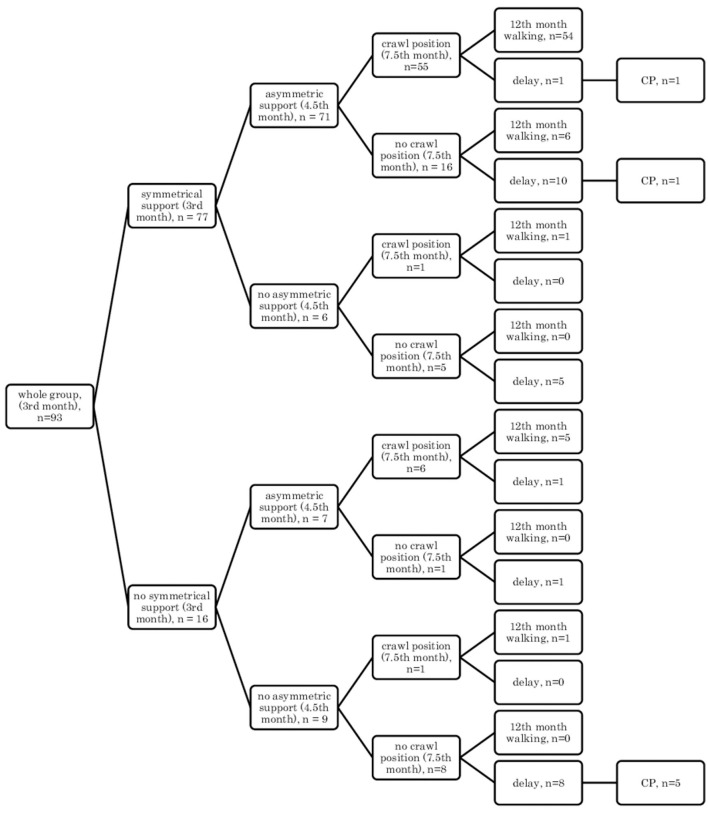
Motor development in the prone position: symmetrical support (3rd month); asymmetric support (4.5th month); crawl position (7.5th month), and independent walking (12th month).

Diagrams, showing the follow-up study of the whole group, are organized according to reaching or not a particular motor skill (quantity assessment) in the following months:

- In the supine position: quadrangle of support (3rd month), turning to the side (4.5th month), creeping (7.5th month), and independent walking (12th month) (Figure 1);
- In the supine position: quadrangle of support (3rd month), turning to the side (4.5th month), crawl position (7.5th month), and independent walking (12th month) (Figure 2);
- In the prone position: symmetrical support (3rd month), asymmetric support (4.5th month), creeping (7.5th month), and independent walking (12th month) (Figure 3);
- In the prone position: symmetrical support (3rd month), asymmetric support (4.5th month), crawl position (7.5th month), and independent walking (12th month) (Figure 4).
- In the prone position: symmetrical support (3rd month), asymmetric support (4.5th month), crawl position (7.5th month), and independent walking (12th month) (Figure 4).

Research showed that if a child, at the age of 3 months, reached the quadrangle of support in the qualitative assessment, then they would mostly demonstrate turning to the side (4.5 months) and they were likely to creep and assume the crawl position, and they would start walking independently at the age of 12 months in the final assessment (57/78 children) Figures 1, 2.

In other cases, when this pattern was not followed, a developmental delay of varying degrees could be observed (in 12/78 children Figure 1 and in 13/78 children, Figure 2).

However, if a child did not reach the quadrangle of support and the dynamics of its development was weak, i.e., it did not turn to the side, it did not creep or did not assume the crawl position, then a delay could be noticed at the age of 12 months, and some of these children at 18 months were diagnosed with CP (5/9 children). In patients with less severe CP (two children: one mild hemiplegia, one mild diplegia), their quantitative development might be normal up to 7.5 months, and independent walking was achieved with a delay after the 12th month of life.

When all children under study have reached 12 months and achieved (or not) turning to the side, creeping, the crawl position, and independent walking, the retrospective analysis of their motor behavior at the age of 3 months was performed. Children were divided into the groups, and the calculation of their motor performance (sum of the points gathered in prone and supine positions) was analyzed. The results are presented in the Tables 1, 2, along with the significance of differences between the subgroups, according to reaching or not a given motor skill (as shown in the diagram), are given in Tables 1, 2. In fact, only three subgroups (of at least seven children) could be analyzed: YES-YES-YES (the best and most numerous), YES-YES-NO (weaker, not reaching the last skill at the age of 7.5 months), and NO-NO-NO (the worst, not acquiring any skill). Children from the NO-NO-NO subgroup were mostly (five out of eight) diagnosed with CP at 18 months. The comparison between the groups was possible only for three of them, the most numerous (as the number of children in other groups were smaller than seven): YES-YES-YES (reached all the investigated milestones on time); YES-YES-NO (reached two earlier milestones, but not independent walking on time); and NO-NO-NO (did not reach any of the expected milestone on time).

**Table 1:** The motor development in supine position: quadrangle of support—turning to the side—creeping OR crawl position, according to the quality of motor performance assessed at the age of 3 months, in prone and supine positions.

Quadrangle of support	YES	YES	YES	YES	NO	NO	NO	NO
turning to the side	YES	YES	NO	NO	YES	YES	NO	NO
creeping	YES	NO	YES	NO	YES	NO	YES	NO
*n* = …	*n* = 60	*n* = 12	*n* = 2	*n* = 4	*n* = 4	*n* = 2	*n* = 0	*n* = 9
Sum of qualitative elements in the prone position	15 (15–15)	15 (13–15)	7, 15	2, 15, 15, 15	2, 6, 6, 7	6, 10	–	6 (0–6)
	6–15	7–15						0–10
Kruskal-Wallis ANOVA and *post-hoc* Dunn's test.								
*H* = 31.58; *p* = 0.000; NO-NO-NO vs. YES-YES-NO and NO-NO-NO vs. YES-YES-YES.								
Sum of qualitative elements in the supine position	15 (15–15)	15 (14–15)	15, 15	0, 15, 15, 15	0, 6, 6, 9	6, 11	–	6 (0–9)
	6–15	7–15						0–11
Kruskal-Wallis ANOVA and *post-hoc* Dunn's test.								
*H* = 32.36; *p* = 0.000; NO-NO-NO vs. YES-YES-NO and NO-NO-NO vs. YES-YES-YES.								
Quadrangle of support	YES	YES	YES	YES	NO	NO	NO	NO
turning to the side	YES	YES	NO	NO	YES	YES	NO	NO
crawl position	YES	NO	YES	NO	YES	NO	YES	NO
*n* = …	*n* = 60	*n* = 12	*n* = 2	*n* = 4	*n* = 4	*n* = 2	*n* = 0	*n* = 9
Sum of qualitative elements in the prone position	15 (15–15)	15 (13–15)	7, 15	15, 15, 15, 15	2, 6, 6, 7	6, 10	–	6 (0–6)
	6–15	7–15						0–10
Kruskal-Wallis ANOVA and *post-hoc* Dunn's test.								
*H* = 31.58; *p* = 0.000; NO-NO-NO vs. YES-YES-NO and NO-NO-NO vs. YES-YES-YES.								
Sum of qualitative elements in the supine position	15 (15–15)	15 (15–15)	15, 15	15, 15, 15, 15	0, 6, 9, 9	6, 11	–	6 (0–6)
	6–15	7–15						0–11
Kruskal-Wallis ANOVA and *post-hoc* Dunn's test.								
*H* = 32.36; *p* = 0.000; NO-NO-NO vs. YES-YES-NO and NO-NO-NO vs. YES-YES-YES.								

Median and quartiles, and min–max are given if the number of children reached at least seven.

**Table 2:** The motor development in the prone position: symmetrical support asymmetric support creeping OR crawl position, according to the quality of motor performance assessed at the age of 3 months, in prone and supine positions.

Symmetrical support	YES	YES	YES	YES	NO	NO	NO	NO
asymmetric support	YES	YES	NO	NO	YES	YES	NO	NO
creeping	YES	NO	YES	NO	YES	NO	YES	NO
*n* = …	*n* = 58	*n* = 13	*n* = 2	*n* = 4	*n* = 6	*n* = 1	*n* = 1	*n* = 8
Sum of qualitative elements in the prone position	15 (15–15)	15 (11–15)	11, 15	6, 15, 15, 15	2, 6, 6, 6, 7, 10	10	7	6 (0–6)
	10–15	7–15						0–8
Kruskal-Wallis ANOVA and *post-hoc* Dunn's test.								
*H* = 32.70; *p* = 0.000; NO-NO-NO vs. YES-YES-NO and NO-NO-NO vs. YES-YES-YES.								
Sum of qualitative elements in the supine position	15 (15–15)	15 (13–15)	12, 15	6, 15, 15, 15	0, 6, 6, 9, 9, 11	11	15	6 (0–9)
	9–15	7–15						0–11
Kruskal-Wallis ANOVA and *post-hoc* Dunn's test.								
*H* = 33.79; *p* = 0.000; NO-NO-NO vs. YES-YES-NO and NO-NO-NO vs. YES-YES-YES.								
Symmetrical support	YES	YES	YES	YES	NO	NO	NO	NO
asymmetric support	YES	YES	NO	NO	YES	YES	NO	NO
crawl position	YES	NO	YES	NO	YES	NO	YES	NO
*n* = ….	*n* = 55	*n* = 16	*n* = 1	*n* = 5	*n* = 6	*n* = 1	*n* = 1	*n* = 8
Sum of qualitative elements in the prone position	15 (15–15)	15 (11–15)	11	6, 15, 15, 15, 15	2, 6, 6, 6, 7, 10	10	7	6 (0–6)
	10–15	7–11						0–8
Kruskal-Wallis ANOVA and *post-hoc* Dunn's test.								
*H* = 32.83; *p* = 0.000; NO-NO-NO vs. YES-YES-NO and NO-NO-NO vs. YES-YES-YES.								
Sum of qualitative elements in the supine position	15 (15–15)	15 (12–15)	12	6, 15, 15, 15, 15	0, 6, 6, 9, 9, 11	11	15	6 (0–9)
	9–15	7–15						0–11
Kruskal-Wallis ANOVA and *post-hoc* Dunn's test.								
*H* = 34.26; *p* = 0.000; NO-NO-NO vs. YES-YES-NO and NO-NO-NO vs. YES-YES-YES.								

Median and quartiles, and min–max are given if the number of children reached at least seven.

Assessment in the prone position showed that if a child achieved symmetrical support on elbows in the quantitative assessment at the age of 3 months, they would mostly show asymmetric support typical of a 4.5-month-old child, and they were highly likely to creep and assume the crawl position (7.5th month), and they would start to walk independently in the final assessment at the age of 12 months (54–56/77 children) Figures 3, 4.

In other cases, when this pattern was not followed, a developmental delay of varying degrees often resulted (15/77 children).

However, if a child did not achieve symmetrical support and the dynamics of their development was weak at a given age, i.e., it did not gain asymmetric support, it did not creep or it did not assume the crawl position, then a delay could be noticed at the age of 12 months, and some of these children were diagnosed with CP at the age of 18 months (5/9 children). Yet, in the case of less severe forms of CP (two children: one mild hemiplegia, one mild diplegia), their quantitative development might be normal up to the age of 7.5 months—they achieved creeping, or in the case of one child—the crawl position. It seems that reaching the crawl position in time is more difficult therefore, it looks more predictive for CP threatening.

Children who obtained the maximum number of points in the qualitative assessment in the supine position in the 3rd month of life (60 children), would turn to the side at the age of 4.5 months and would start to creep and assume the crawl position at 7.5 months. The probability of walking on time was 95%.

An analysis of qualitative development in the supine position showed that when a child did not score the maximum number of points at the age of 3 months, it could perform the quadrangle of support and turn to the side on time. Still, it was very likely not to creep or assume the crawl position (Table 1).

Children who obtained the maximum number of points in the qualitative assessment in the prone position in the 3rd month of life (58 children) would perform the asymmetric support at the age of 4.5 months, and would start to creep and assume the crawl position and, with 96–98% probability, would start to walk at the age of 12 months (Table 2).

The assessment of qualitative development in the prone position showed that when a child did not score the maximum number of points in the 3rd month of life, it could perform symmetrical support on time and then asymmetric support. It has been proven that children from this group would not creep, and most of them would not assume the crawl position on time.

Children who failed to achieve any skill could still score a few points in the qualitative assessment at 3 months, but this applied to distal features. However, all children diagnosed with more severe CP did not score any points in the qualitative assessment, neither supine nor prone.

One can notice so-called exceptions during the analysis, i.e., children who, in the quantitative assessment, up to the point of achieving creeping or even the crawl position in one child, developed correctly. This is true for two children with a mild form of CP. Therefore, for the early detection of motor disorders, a qualitative assessment is needed: these two children were assessed to score 11 and 10 points in the prone position out of 15 possible at the age of 3 months, and 11 and 9 points in the supine position out of 15 possible, respectively. The total high score, especially in the prone position, guarantees the achievement of the basic analyzed milestones: symmetrical support—asymmetric support—creeping.

In the qualitative assessment for the age of 3 months, as outlined in the test procedure, one of the components is the assessment of pelvic alignment. In our study, both the assessment in the supine and prone position in the 3rd month of life showed that in children with CP, also in those two with a mild form, the pelvis was positioned incorrectly (18).

It is worth noting that development in most children is typical, and the expected motor skills are achieved on time. This is illustrated by the following: in the supine position: a quadrangle of support—turning to the side—creeping or crawl position; in the prone position, respectively: symmetrical support—asymmetric support—creeping or crawl position. These children will also be able to walk independently on time.

On the other hand, if the development at the age of 3 months is no longer normal (absence of a quadrangle of support or symmetrical support), the achievement of following skills will probably be delayed. There will be exceptions to such a very general rule, disturbances may occur at each stage, but they are individual. One must remember that the assessment takes place at a given moment, and the child may have acquired a specific skill a little later. For the study, fixed time points were adopted in accordance with the literature, which does not exclude the possibility that a given skill would appear later.

## Discussion

We wanted to show (by analyzing children from one cultural area) how normal or abnormal motor development at the age of 3 months is crucial for reaching milestones and that it can be an early warning sign for children at risk of CP.

The findings of our study have shown that achieving appropriate motor skills in the prone position is more difficult. Still, it is a better guarantee of proper motor development in the 12th month of life (56 out of 58 children, i.e., 96%), and that the crawl position is more difficult to achieve than the creeping, but it is also more predictive (54/55, i.e., 98%).

The varied nature of the nervous system and its constant interaction with different environments cause a significant variation in motor development between individual children. Motor development is characterized by the diversification of the way of performing tasks and intra- and inter-individual variants of the pace of achieving milestones. We realize that, as a result, the age at which motor milestones are achieved is very scattered—also across cultures (1). However, to be able to assess a child's motor development, it is necessary to adopt the age frame for independent achievement of specific motor activities, as is the case in our study. We also see that failure to achieve one assessed motor activity does not mean a diagnosis of CP in the future.

It can also be seen that there are always cases of atypical development disorders, but they are few (incidental) concerning the entire group because the most numerous courses were YES-YES-YES; YES-YES-NO; and NO-NO-NO. More deviations from the proper course and the atypical course (e.g., NO-YES-YES or YES-NO-NO) apply to the test in the prone position. Most babies who reached the relevant milestones on time would achieve normal motor development by 12 months, i.e., independent walking. This was found in the case of assessment in the prone position, 56/77 (73%; Figure 3) and 54/77 (70%, Figure 4), and 57/78 (73%, Figure 1) and 57/78 (73%, Figure 2) in the case of assessment in the supine position, respectively. This thesis that the rate at which certain motor skills can be achieved may vary and that the child's delayed display of a single milestone is of limited clinical value confirms the findings by other researchers. However, a delay in reaching multiple milestones suggests an increased risk of developmental pathology (23).

This was confirmed in a small group of five children who showed a motor deficit from the beginning, and eventually—at the age of 18 months they were diagnosed with CP. It is worth emphasizing that this abnormal development can be detected already in the 3rd month of life and that these children should undergo rehabilitation immediately after seeing deficits.

In the case of mild neurological disorders (a mild form of CP), it is difficult to detect abnormalities with 100% probability. Still, in the qualitative assessment at 3 months, these children never scored the maximum number of points. This suggests that children who, in the qualitative assessment at the age of 3 months, were found to have even minor abnormalities should remain under observation, and the abnormalities should be eliminated as soon as possible through properly conducted rehabilitation. The data analysis showed that, with a high probability, even these slight abnormalities indicate the occurrence of developmental delay and even neurological disorders (a mild form of CP).

It was interesting for us which element of the qualitative assessment was predictive of further development in the study group. In previous articles (18, 24), we proved that the pelvis position is essential in detecting disorders such as developmental delay or diagnosis CP. We also observed similar relationships in the analyzed studies.

Novak et al. presented, through the analysis of the literature, the tools which, before the age of 5 months, feature high predictive validity in early diagnosis of CP. These include MRI (86–89% sensitivity), General Movements (GMs) 98% sensitivity and the HINE 90% sensitivity, and after the age of 5 months, GMs is replaced by the Developmental Assessment of Young Children (83 % sensitivity) (25). As described in previous articles, these tools are very sensitive but of little use to apply early physiotherapeutic intervention (18, 22). For a physiotherapeutic assessment, a sensitive, reliable, simple, and valuable tool is essential, i.e., one that makes it possible to set therapeutic goals uniformly and measure changes resulting from the therapy.

## Study limitation

This study was not intended as population research (screening) but was performed on the cohort of children with some suspicious motor behaviors. The number of children is thus limited and the number of suspected CP relatively higher than in general population (9 out of 93, i.e., 10% instead of 1–2%). Nevertheless, we think that even a larger group of normally developing children would show the same pattern of motor behavior along with time.

## Conclusion

A correct qualitative assessment in the 3rd month of life guarantees a high probability of the appearance of turning to the side and asymmetric support at the age of 4.5 months, crawl position and creeping at 7.5 months, and walking at 12 months. If the qualitative assessment in the 3rd month is very low or even zero, it is very likely that CP will be diagnosed at the age of 18 months. Children with a low quantitative assessment and poorly positioned pelvis may develop a mild form of CP.

## Data availability statement

The data analyzed in this study is subject to the following licenses/restrictions: The dataset are available for the Editor if it is necessary. Requests to access these datasets should be directed to EG ewagajewska1011@gmail.com.

## Ethics statement

The study was approved by the Research Ethics Committee of Poznan University of Medical Sciences and registered under no. 22/10 (07-01-2010). Written informed consent to participate in this study was provided by the participant's legal guardian/next of kin.

## Author contributions

EG and MS contributed to the conceptualization, formal analysis, resources, and writing—original draft. EG contributed to the data curation and funding acquisition. MS contributed to the methodology. EG and MN contributed to the project administration. EG, MN, AN, and MS contributed to the investigation and writing—review and editing. All authors have read and agreed to the published version of the manuscript.

## Conflict of interest

The authors declare that the research was conducted in the absence of any commercial or financial relationships that could be construed as a potential conflict of interest.

## Publisher's note

All claims expressed in this article are solely those of the authors and do not necessarily represent those of their affiliated organizations, or those of the publisher, the editors and the reviewers. Any product that may be evaluated in this article, or claim that may be made by its manufacturer, is not guaranteed or endorsed by the publisher.
